# Shear Instability Control of Hybrid Small-Scale Plates Embedded in a Polymer Matrix via Shape Memory Alloy Nanofibers

**DOI:** 10.3390/mi17030295

**Published:** 2026-02-27

**Authors:** Mohammad Reza Farajpour, Mohammad Danesh, Mohammad Hossein Shokrani, Ali Farajpour

**Affiliations:** 1 Department of Mechanical Engineering, Isfahan University of Technology, Isfahan 8415683111, Iran; mfarajpour68@gmail.com (M.R.F.); danesh@iut.ac.ir (M.D.); mhosein.shokrani@gmail.com (M.H.S.); 2 The Queen Elizabeth Hospital, Woodville South, SA 5011, Australia; 3 School of Medicine, Robinson Research Institute, College of Health, Adelaide University, Adelaide, SA 5005, Australia

**Keywords:** instability control, small-scale plates, SMA nanofibers, shear capacity, modified elasticity

## Abstract

A refined mathematical framework is developed to investigate the ability of shape memory alloy (SMA) nanofibers to control the shear instability of a hybrid small-scale plate made of three layers containing nanofibers. The middle layer is reinforced by SMA nanofibers, while typical nanofibers are utilized to reinforce other layers. Using the Brinson theory, the nonlocal theory and the principle of virtual work, the scale-dependent coupled equations of the reinforced ultrasmall plate are presented. A differential quadrature technique is then applied as a solution procedure for different edge conditions. The influences of various factors, including the coefficients of the polymer matrix, the recovery stress, orientation and volume fraction of SMA nanofibers on the control ability are studied. It is concluded that the shear instability capacity of small-scale plates can be reasonably controlled by using SMA nanofibers. Particularly, higher recovery stresses result in higher critical shear loads. As the SMA volume fraction increases, the shear instability load remarkably increases.

## 1. Introduction

Fundamental smart nanoscale structures such as piezoelectric nanobeams, magneto-electro-elastic nanobeams and nanoplates, as well as shape memory alloy (SMA) nanofibers, have received researchers’ interest since they exhibit excellent mechanical and electric properties [1,2,3]. Particularly, SMA nanofibers have the potential to be used in nanoelectromechanical systems (NEMS) such as nanosensors and nanoactuators in order to improve the electromechanical response of these valuable systems [4,5]. In addition, these small-scale structures are promising candidates for use in microelectromechanical systems (MEMS) [6,7] for energy harvesting, sensing and power generation.

The rapid advancement of small-scale electromechanical devices has led to a key requirement for structural elements capable of mechanical resistance under different loading conditions [8,9]. In recent years, hybrid small-scale plates reinforced with nanofibers have been in the centre of attention because they exhibit a high stiffness-to-weight ratio and adjustable mechanical characteristics [10,11]. At the small-scale levels, such as nanoscales and microscales, the mechanical characteristics of materials are highly dependent on their size and thus, their accurate modelling requires a size-dependent simulation approach [12,13]. Among different loading conditions, shear forces are of particular importance since this loading can cause sudden instability and serious structural damage. Shape memory alloys (SMAs), because of their unique recovery stresses and adaptive stiffness responses, hold great promise for use as a tool to control the shear instability when implemented as nanofibers. The capacity to improve resistance to structural instability is critical in many applications, including MEMS/NEMS devices, biomedical small-scale systems, flexible electromechanical devices and smart adaptive structures, where enhanced loading capacity is essential.

The response of both smart and typical nanomaterials, such as carbon nanotubes, nanowires, graphene sheets, piezoelectric nanoplates and SMA nanofibers to an external stimulus depends on their size [14,15,16,17]. It is essential to incorporate scale effects into the theoretical modelling of these nanomaterials [18,19,20]. To this end, modified advanced theories, including, but not limited to, couple stress [21,22], strain gradient [23,24], nonlocal [25,26], and nonlocal strain gradient [27,28] models, are widely employed. The nonlocal elasticity-based model is applied in the current investigation due to its simplicity and capability [29,30].

A review study on a couple of closely relevant articles is given to illuminate the state of the art of scale-dependent modelling for an interested reader. Malekzadeh et al. [31] employed a nonlocal model to describe the impact of size effects on the vibrations of skew nanoplates. In another valuable work, Malekzadeh and Shojaee [32] studied the vibration of rectangular nanoplates based on a combination of the nonlocal plate theory and a two-variable deformation theory. Aksencer and Aydogdu [33] developed a nonlocal continuum model for exploring the forced vibration of plates at nanoscale levels. Moreover, Aydogdu [34] proposed a scale-dependent rod model so as to study the longitudinal vibration of a double-walled carbon nanotube (CNTs); he took into account the effect of axial van der Waals forces on the vibrational behaviour. In another investigation, a nonlocal beam model was developed for exploring the wave propagation in CNTs along their axial direction [35]. Farajpour et al. [36] studied the dynamic response of smart reinforced ultra-small films for mass sensor applications in the context of a nonlocal theory. Zenkour [37] developed a scale-dependent model to study the thermo-mechanical instability of carbon nanotubes on a viscoelastic foundation. Gul and Aydogdu [38] developed a higher-order doublet mechanics model for analyzing flexural waves propagated within single-walled and double-walled CNTs based on the Timoshenko theory of beams. Their results demonstrated the important role of scale effects in the flexural wave propagation characteristics of CNTs.

The scale-dependent oscillations and instability of small-scale structures with non-homogeneous material properties have been investigated [39,40]. Nejad and his co-workers [41] developed an Euler–Bernoulli model of stress nonlocality to study the nanomechanical instability of arbitrary two-directional functionally graded beams at nanoscale levels. In a relevant research work, a nonlocal model was also proposed for the vibrational response of bi-directional functionally graded nanobeams [42]. Barati et al. [43] examined the effects of a magnetic field on the transverse vibrational behaviour of bi-directional functionally graded nanobeams. They concluded that a magnetic field can be utilized to adjust and control the vibration response of non-homogeneous structures at nanoscales.

Size-dependent models of elasticity have been used to predict the biomechanics of small-scale biological structural components, especially protein microtubules [44]. These biomechanical features include, but are not limited to, vibrational response [45], buckling behaviour [46], static deformation (persistence lengths) [47], dynamic stability [48] and wave propagation [49]. In addition, scale-dependent modified elasticity theories have been used to analyze hybrid bioinspired structures such as CNT-microtubule composite nanotubes enhanced by a magneto-electro-elastic layer [50] and porous sandwich bionanoplates composed of functionally graded materials and lipid bilayers [51,52].

Liu et al. [53] presented a nonlocal plate theory so as to examine the linear thermo-electro-mechanical oscillations of nanoscale plates with piezoelectric properties; they obtained the scale-dependent natural frequencies of simply supported piezoelectric nanoscale plates using an analytical method. In addition, a size-dependent plate theory was introduced in Ref. [54] for describing size influences on the vibrational behaviour of a smart nanoscale system made of two piezoelectric ultrasmall plates. A two-dimensional linear size-dependent model was also proposed by Wang et al. [55] to incorporate scale effects on the mechanical behaviour of magneto-electro-elastic micro/nanoscale plates. The effect of edge displacements on the vibration and stability of magneto-electro-elastic nanoplates was studied via the nonlocal elasticity [56]. More recently, Zhang and Li have [57] developed an advanced size-dependent model for the mechanics of spherical nanoscale particles. They incorporated both stress nonlocality and surface interactions into their model. They also demonstrated the validity of nonlocal continuum mechanics using molecular statics simulations and experimental data for nanoscale problems, including the mechanical behaviour of nanoparticles [57]. Li et al. [58] proposed a spatiotemporally nonlocal strain gradient model for assessing the microstructural dependency and history dependency of interconnected transient polymer networks using a thermodynamics approach. In a relevant research work, a nonlocal strain gradient theory has been developed for crosslinked polymers [59].

Compared to well-known nanostructural elements such as graphene sheets and CNTs, the size-dependent mechanical characteristics of SMA nanofiber-reinforced hybrid ultrasmall structures have not been investigated thoroughly. While there are some previous research works on the buckling response and vibrational behaviour of SMA-reinforced nanoscale plates [20,60], the main focus of the present study is on shear instability rather than traditional compressive buckling or free vibration, where no external forces are applied. In many practical situations, the building blocks of NEMS-based devices are likely to be under the action of a shear loading instead of normal loads. Therefore, it is important to enhance the level of knowledge of the mechanical response of NEMS-based systems subject to shear loads. On the other hand, to improve the total stiffness of nano-structural elements, they are commonly embedded in a polymer matrix or a flexible substrate in realistic operating scenarios. To the best of our knowledge, the influence of shear forces, coupled with nonlocal interactions and polymer matrix effects, has not yet been investigated on the instability response of SMA nanofiber-reinforced hybrid ultrasmall plates. Furthermore, in this study, emphasis is put on the practical use case of SMA nanofibers, where their recovery stress and the surrounding polymer matrix or substrate can be used to control the instability behaviour of a hybrid small-scale system. This embedded layered hybrid design helps targeted instability control, which is more challenging in single-layer reinforced configurations.

In the present paper, for the first time, a modified model is developed to assess the ability of SMA nanofibers to provide a platform for controlling the shear instability of hybrid ultrasmall plates embedded in a polymer matrix. The Eringen theory of elasticity is utilized to take into account scale effects. The influence of the polymer matrix is modelled on the basis of the Pasternak foundation model. Using Brinson’s theory and the nonlocal elasticity, the coupled scale-dependent equations of hybrid ultrasmall plates reinforced by SMA nanofibers are obtained. The differential quadrature (DQ) approach as a numerical tool is applied to the derived coupled equations. Small-scale plates with different edge conditions, such as clamped and simply supported, are taken into account. It is anticipated that the present model would be helpful in the smart control of NEMS-based devices using SMA nanofibers.

## 2. Size-Dependent Formulation

**Figure 1:**
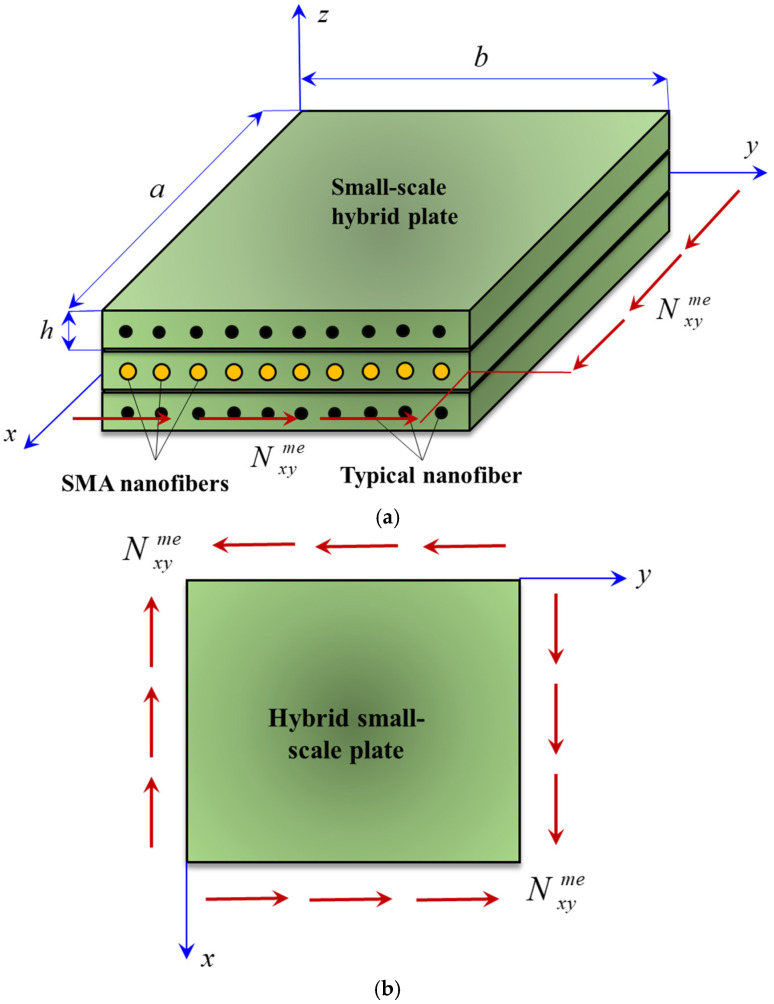
(**a**) Controlling the stability of a hybrid small-scale plate using SMA nanofibers; (**b**) the applied shear in-plane loading.

In Figure 1, a typical hybrid small-scale plate reinforced by SMA nanofibers under the action of a shear in-plane loading (i.e., *N_xy_*) is indicated, where *a*, *b* and *h* denote the length, width and thickness of every nanoplate, respectively. The hybrid plate is composed of three elastic layers and bundles of typical and SMA nanofibers. The top and bottom layers are reinforced by typical nanofibers, while SMA nanofibers are used to reinforce the middle layer. According to Brinson’s model, one can write

(1) $$\Upsilon =\Upsilon_{S}+\Upsilon_{T},$$

in which Υ, $\Upsilon_{S}$ and $\Upsilon_{T}$ represent the total, mechanical stress-induced and temperature-induced martensite fractions, respectively.

The following relation between the total martensite fraction and the elasticity modulus (*E*) can be employed for SMA nanofibers.

(2) $$E(\Upsilon )=\frac{E_{M}}{\left(1-\Upsilon \right)\left(E_{M}/E_{A}\right)+\Upsilon }.$$

In the above relation, *E_M_* and *E_A_* stand for Young’s modulus in pure martensite and austenite phases, respectively. On the other hand, the relation between the martensite fractions and the temperature (*T*) as well as the mechanical stress (σ) for the martensite-to-austenite conversion can be expressed as [61]

(3) $$\Upsilon =0.5\Upsilon_{0}\cos\left[\Xi (T,\sigma )\right]+0.5\Upsilon_{0},$$

(4) $$\Xi =\frac{\pi \left(T-A_{s}\right)}{A_{f}-A_{s}}-\frac{\pi \sigma }{C_{A}\left(A_{f}-A_{s}\right)},$$

(5) $$\left\{\begin{array}{c} \Upsilon_{T}\\ \Upsilon_{S} \end{array}\right\}=\left\{\begin{array}{c} \Upsilon_{T0}\\ \Upsilon_{S0} \end{array}\right\}-\frac{1}{\Upsilon_{0}}\left\{\begin{array}{c} \Upsilon_{T0}\\ \Upsilon_{S0} \end{array}\right\}\left(\Upsilon_{0}-\Upsilon \right),\;$$

in which *T* > *A_S_* and *C_A_*(*T* − *A_f_*) <  σ  *< C_A_*(*T* − *A_S_*); ‘0’ denotes the initial values of each parameter. *A_s_* and *A_f_* are respectively the start and finish temperatures for the austenite phase; *C_A_* is the critical stress slope for the martensite-to-austenite conversion. The constitutive equation of SMA nanofibers is as follows

(6) $$\sigma =E(\Upsilon )\varepsilon -\Upsilon_{S}E(\Upsilon )\varepsilon_{\max}+\alpha \Delta T.$$

Here ε and $\varepsilon_{max}$ are respectively the strain and the maximum residual strain of the nanofiber; α and ΔT are also the thermal coefficient and the temperature change from the reference temperature, respectively. Now, let us take into account a hybrid plate reinforced by SMA nanofibers of volume fraction $V_{nf}^{*}$. The mechanical properties of the hybrid plate can be calculated by [62]

(7) $$E_{1}\left(\Upsilon \right)=E_{np}+\left(E_{nf}-E_{np}\right)V_{nf}^{*}\left(\Upsilon \right),\;E_{2}\left(\Upsilon \right)=\frac{E_{np}}{1+\left(\left(E_{np}-E_{nf}\right)/E_{nf}\right)V_{nf}^{*}\left(\Upsilon \right)},\;\nu_{12}\left(\Upsilon \right)=\nu_{np}+\left(\nu_{nf}-\nu_{np}\right)V_{nf}^{*}\left(\Upsilon \right),\;G_{12}\left(\Upsilon \right)=\frac{G_{np}}{1+\left(\left(G_{np}-G_{nf}\right)/G_{nf}\right)V_{nf}^{*}\left(\Upsilon \right)},$$

where ‘*np*’ and ‘*nf*’ indicate nanoplates and nanofibers, respectively. *G_ij_*, *v_ij_* and *E_i_* represent the shear modulus, Poisson’s ratio and Young’s modulus, respectively. The strain-displacement relations for the hybrid nanoplate are as

(8) $$\left\{\begin{array}{c} \varepsilon_{xx}\\ \varepsilon_{yy}\\ \gamma_{xy} \end{array}\right\}=\left\{\begin{array}{c} \frac{\partial u}{\partial x}\\ \frac{\partial v}{\partial y}\\ \frac{\partial u}{\partial y}+\frac{\partial v}{\partial x} \end{array}\right\}-z\left\{\begin{array}{c} \frac{\partial^{2}w}{\partial x^{2}}\\ \frac{\partial^{2}w}{\partial y^{2}}\\ 2\frac{\partial^{2}w}{\partial x\partial y} \end{array}\right\},$$

where *u*, *v* and *w* stand for the displacement components along the *x*, *y* and *z* axes, respectively. Based on the Eringen theory of elasticity, the stress–strain relation of the *i*th layer of the hybrid nanoplate can be formulated as

(9) $$\begin{array}{l} \left[1-{\left(e_{0}l_{c}\right)}^{2}\nabla^{2}\right]\left\{\begin{array}{c} \sigma_{xx}^{\left(i\right)}\\ \sigma_{yy}^{\left(i\right)}\\ \sigma_{xy}^{\left(i\right)} \end{array}\right\}=\left[\begin{array}{ccc} {\bar{C}}_{11}^{\left(i\right)}(\Upsilon ) & {\bar{C}}_{12}^{\left(i\right)}(\Upsilon ) & {\bar{C}}_{16}^{\left(i\right)}(\Upsilon )\\ {\bar{C}}_{12}^{\left(i\right)}(\Upsilon ) & {\bar{C}}_{22}^{\left(i\right)}(\Upsilon ) & {\bar{C}}_{26}^{\left(i\right)}(\Upsilon )\\ {\bar{C}}_{16}^{\left(i\right)}(\Upsilon ) & {\bar{C}}_{26}^{\left(i\right)}(\Upsilon ) & {\bar{C}}_{66}^{\left(i\right)}(\Upsilon ) \end{array}\right]\left\{\begin{array}{c} \varepsilon_{xx}\\ \varepsilon_{yy}\\ \gamma_{xy} \end{array}\right\}\\ +\left\{\begin{array}{c} {\overset{⌣}{C}}^{2}\left(\psi_{i}\right)\\ {\overset{⌣}{S}}^{2}\left(\psi_{i}\right)\\ \overset{⌣}{S}\left(\psi_{i}\right)\overset{⌣}{C}\left(\psi_{i}\right) \end{array}\right\}V_{nf,i}^{*}\sigma_{r}^{\left(i\right)}, \end{array}$$

where

(10) $$\begin{array}{l} {\bar{C}}_{11}^{\left(i\right)}(\Upsilon )=C_{11}^{\left(i\right)}(\Upsilon ){\overset{⌣}{C}}^{4}(\psi_{i})+C_{22}^{\left(i\right)}(\Upsilon ){\overset{⌣}{S}}^{4}(\psi_{i})\\ +2\left[C_{12}^{\left(i\right)}(\Upsilon )+2C_{66}^{\left(i\right)}(\Upsilon )\right]{\overset{⌣}{S}}^{2}(\psi_{i}){\overset{⌣}{C}}^{2}(\psi_{i}),\\ {\bar{C}}_{12}^{\left(i\right)}(\Upsilon )=\left[C_{11}^{\left(i\right)}(\Upsilon )+C_{22}^{\left(i\right)}(\Upsilon )-4C_{66}^{\left(i\right)}(\Upsilon )\right]{\overset{⌣}{S}}^{2}(\psi_{i}){\overset{⌣}{C}}^{2}(\psi_{i})\\ +C_{12}^{\left(i\right)}(\Upsilon )\left[{\overset{⌣}{C}}^{4}(\psi_{i})+{\overset{⌣}{S}}^{4}(\psi_{i})\right],\\ {\bar{C}}_{22}^{\left(i\right)}(\Upsilon )=C_{22}^{\left(i\right)}(\Upsilon ){\overset{⌣}{C}}^{4}(\psi_{i})+C_{11}^{\left(i\right)}(\Upsilon ){\overset{⌣}{S}}^{4}(\psi_{i})\\ +2\left[C_{12}^{\left(i\right)}(\Upsilon )+2C_{66}^{\left(i\right)}(\Upsilon )\right]{\overset{⌣}{S}}^{2}(\psi_{i}){\overset{⌣}{C}}^{2}(\psi_{i}),\\ {\bar{C}}_{16}^{\left(i\right)}(\Upsilon )=\left[C_{11}^{\left(i\right)}(\Upsilon )-C_{12}^{\left(i\right)}(\Upsilon )-2C_{66}^{\left(i\right)}(\Upsilon )\right]\overset{⌣}{S}(\psi_{i}){\overset{⌣}{C}}^{3}(\psi_{i})\\ -\left[C_{22}^{\left(i\right)}(\Upsilon )-C_{12}^{\left(i\right)}(\Upsilon )-2C_{66}^{\left(i\right)}(\Upsilon )\right]{\overset{⌣}{S}}^{3}(\psi_{i})\overset{⌣}{C}(\psi_{i}),\\ {\bar{C}}_{26}^{\left(i\right)}(\Upsilon )=\left[C_{11}^{\left(i\right)}(\Upsilon )-C_{12}^{\left(i\right)}(\Upsilon )-2C_{66}^{\left(i\right)}(\Upsilon )\right]{\overset{⌣}{S}}^{3}(\psi_{i})\overset{⌣}{C}(\psi_{i})\\ -\left[C_{22}^{\left(i\right)}(\Upsilon )-C_{12}^{\left(i\right)}(\Upsilon )-2C_{66}^{\left(i\right)}(\Upsilon )\right]\overset{⌣}{S}(\psi_{i}){\overset{⌣}{C}}^{3}(\psi_{i}),\\ {\bar{C}}_{66}^{\left(i\right)}(\Upsilon )=\left[C_{11}^{\left(i\right)}(\Upsilon )+C_{22}^{\left(i\right)}(\Upsilon )-2C_{12}^{\left(i\right)}(\Upsilon )-2C_{66}^{\left(i\right)}(\Upsilon )\right]{\overset{⌣}{S}}^{2}(\psi_{i}){\overset{⌣}{C}}^{2}(\psi_{i})\\ +C_{66}^{\left(i\right)}(\Upsilon )\left[{\overset{⌣}{S}}^{4}(\psi_{i})+{\overset{⌣}{C}}^{4}(\psi_{i})\right], \end{array}$$

and

(11) $$\begin{array}{l} \left\{\begin{array}{c} C_{11}(\Upsilon )\\ C_{22}(\Upsilon ) \end{array}\right\}=\frac{1}{\overset{⌣}{\nu }(\Upsilon )}\left\{\begin{array}{c} E_{1}(\Upsilon )\\ E_{2}(\Upsilon ) \end{array}\right\},\\ \left\{\begin{array}{c} C_{12}(\Upsilon )\\ \;C_{66}(\Upsilon ) \end{array}\right\}=\frac{\nu_{21}(\Upsilon )}{\overset{⌣}{\nu }(\Upsilon )}\left\{\begin{array}{c} E_{1}(\Upsilon )\\ \frac{\overset{⌣}{\nu }(\Upsilon )}{\nu_{21}(\Upsilon )}G_{12}(\Upsilon ) \end{array}\right\}, \end{array}$$

Here $\overset{˘}{C}(\psi_{i})=cos(\psi_{i})$, $\overset{˘}{S}(\psi_{i})=sin(\psi_{i})$ and $\overset{˘}{\nu }(\Upsilon )=1-\nu_{12}(\Upsilon )\nu_{21}(\Upsilon )$. In Equation (9), $l_{c}$ denotes an internal characteristic length for the hybrid nanosystem, and *e*_0_ is a calibration parameter; $\psi_{i}$ and $\sigma_{r}^{\left(i\right)}$ are respectively the nanofiber angle and the recovery stress of the *i*th plate; the Laplace operator is denoted as $\nabla^{2}$. Employing Equations (8) and (9), the stress resultants of the hybrid nanosystem are obtained as

(12) $$\begin{array}{l} \left[1-{\left(e_{0}l_{c}\right)}^{2}\nabla^{2}\right]N_{xx}={\tilde{K}}_{11}\frac{\partial u}{\partial x}+{\tilde{K}}_{12}\frac{\partial v}{\partial y}+{\tilde{K}}_{16}\left(\frac{\partial u}{\partial y}+\frac{\partial v}{\partial x}\right)\\ -{\tilde{Q}}_{11}\frac{\partial^{2}w}{\partial x^{2}}-{\tilde{Q}}_{12}\frac{\partial^{2}w}{\partial y^{2}}-2{\tilde{Q}}_{16}\frac{\partial^{2}w}{\partial x\partial y}+N_{xx}^{r},\\ \left[1-{\left(e_{0}l_{c}\right)}^{2}\nabla^{2}\right]N_{yy}={\tilde{K}}_{12}\frac{\partial u}{\partial x}+{\tilde{K}}_{22}\frac{\partial v}{\partial y}+{\tilde{K}}_{26}\left(\frac{\partial u}{\partial y}+\frac{\partial v}{\partial x}\right)\\ -{\tilde{Q}}_{12}\frac{\partial^{2}w}{\partial x^{2}}-{\tilde{Q}}_{22}\frac{\partial^{2}w}{\partial y^{2}}-2{\tilde{Q}}_{26}\frac{\partial^{2}w}{\partial x\partial y}+N_{yy}^{r},\\ \left[1-{\left(e_{0}l_{c}\right)}^{2}\nabla^{2}\right]N_{xy}={\tilde{K}}_{16}\frac{\partial u}{\partial x}+{\tilde{K}}_{26}\frac{\partial v}{\partial y}+{\tilde{K}}_{66}\left(\frac{\partial u}{\partial y}+\frac{\partial v}{\partial x}\right)\\ -{\tilde{Q}}_{16}\frac{\partial^{2}w}{\partial x^{2}}-{\tilde{Q}}_{26}\frac{\partial^{2}w}{\partial y^{2}}-2{\tilde{Q}}_{66}\frac{\partial^{2}w}{\partial x\partial y}+N_{xy}^{r}, \end{array}$$

(13) $$\begin{array}{l} \left[1-{\left(e_{0}l_{c}\right)}^{2}\nabla^{2}\right]M_{xx}={\tilde{Q}}_{11}\frac{\partial u}{\partial x}+{\tilde{Q}}_{12}\frac{\partial v}{\partial y}+{\tilde{Q}}_{16}\left(\frac{\partial u}{\partial y}+\frac{\partial v}{\partial x}\right)\\ -{\tilde{S}}_{11}\frac{\partial^{2}w}{\partial x^{2}}-{\tilde{S}}_{12}\frac{\partial^{2}w}{\partial y^{2}}-2{\tilde{S}}_{16}\frac{\partial^{2}w}{\partial x\partial y}+M_{xx}^{r},\\ \left[1-{\left(e_{0}l_{c}\right)}^{2}\nabla^{2}\right]M_{yy}={\tilde{Q}}_{12}\frac{\partial u}{\partial x}+{\tilde{Q}}_{22}\frac{\partial v}{\partial y}+{\tilde{Q}}_{26}\left(\frac{\partial u}{\partial y}+\frac{\partial v}{\partial x}\right)\\ -{\tilde{S}}_{12}\frac{\partial^{2}w}{\partial x^{2}}-{\tilde{S}}_{22}\frac{\partial^{2}w}{\partial y^{2}}-2{\tilde{S}}_{26}\frac{\partial^{2}w}{\partial x\partial y}+M_{yy}^{r},\\ \left[1-{\left(e_{0}l_{c}\right)}^{2}\nabla^{2}\right]M_{xy}={\tilde{Q}}_{16}\frac{\partial u}{\partial x}+{\tilde{Q}}_{26}\frac{\partial v}{\partial y}+{\tilde{Q}}_{66}\left(\frac{\partial u}{\partial y}+\frac{\partial v}{\partial x}\right)\\ -{\tilde{S}}_{16}\frac{\partial^{2}w}{\partial x^{2}}-{\tilde{S}}_{26}\frac{\partial^{2}w}{\partial y^{2}}-2{\tilde{S}}_{66}\frac{\partial^{2}w}{\partial x\partial y}+M_{xy}^{r}, \end{array}$$

where

(14) $${\tilde{K}}_{ij}=\sum_{k=1}^{n}{\bar{C}}_{ij}^{\left(k\right)}\left(z_{k}-z_{k-1}\right),\;{\tilde{Q}}_{ij}=\frac{1}{2}\sum_{k=1}^{n}{\bar{C}}_{ij}^{\left(k\right)}\left(z_{k}^{2}-z_{k-1}^{2}\right),\;{\tilde{S}}_{ij}=\frac{1}{3}\sum_{k=1}^{n}{\bar{C}}_{ij}^{\left(k\right)}\left(z_{k}^{3}-z_{k-1}^{3}\right).$$

Here *n* is the number of layers. The stress resultants are defined as

(15) $$\left\{\begin{array}{c} N_{xx}\\ N_{yy}\\ N_{xy} \end{array}\right\}=\sum_{i=1}^{n}\int_{z_{i-1}}^{z_{i}}\left\{\begin{array}{c} \sigma_{xx}^{\left(i\right)}\\ \sigma_{yy}^{\left(i\right)}\\ \sigma_{xy}^{\left(i\right)} \end{array}\right\}dz,\;\;\left\{\begin{array}{c} M_{xx}\\ M_{yy}\\ M_{xy} \end{array}\right\}=\sum_{i=1}^{n}\int_{z_{i-1}}^{z_{i}}\left\{\begin{array}{c} \sigma_{xx}^{\left(i\right)}\\ \sigma_{yy}^{\left(i\right)}\\ \sigma_{xy}^{\left(i\right)} \end{array}\right\}zdz.$$

In addition, the recovery stress-induced force and moment resultants are defined as

(16) $$\left\{\begin{array}{c} N_{xx}^{r}\\ N_{yy}^{r}\\ N_{xy}^{r} \end{array}\right\}=\sum_{i=1}^{n}\int_{z_{i-1}}^{z_{i}}V_{nf,i}^{*}\sigma_{r}^{\left(i\right)}\left\{\begin{array}{c} {\overset{⌣}{C}}^{2}\left(\psi_{i}\right)\\ {\overset{⌣}{S}}^{2}\left(\psi_{i}\right)\\ \overset{⌣}{S}\left(\psi_{i}\right)\overset{⌣}{C}\left(\psi_{i}\right) \end{array}\right\}dz,\;\left\{\begin{array}{c} M_{xx}^{r}\\ M_{yy}^{r}\\ M_{xy}^{r} \end{array}\right\}=\sum_{i=1}^{n}\int_{z_{i-1}}^{z_{i}}V_{nf,i}^{*}\sigma_{r}^{\left(i\right)}\left\{\begin{array}{c} {\overset{⌣}{C}}^{2}\left(\psi_{i}\right)\\ {\overset{⌣}{S}}^{2}\left(\psi_{i}\right)\\ \overset{⌣}{S}\left(\psi_{i}\right)\overset{⌣}{C}\left(\psi_{i}\right) \end{array}\right\}zdz.$$

It is assumed that the SMA nanofiber-reinforced hybrid nanoplate is embedded in a polymer matrix. According to the virtual work principle and using the Pasternak foundation model, one obtains

(17) $$\frac{\partial N_{xy}}{\partial y}=-\frac{\partial N_{xx}}{\partial x},$$

(18) $$\frac{\partial N_{xy}}{\partial x}=-\frac{\partial N_{yy}}{\partial y},$$

(19) $$\frac{\partial^{2}M_{xx}}{\partial x^{2}}+\frac{\partial^{2}M_{yy}}{\partial y^{2}}+2\frac{\partial^{2}M_{xy}}{\partial y\partial x}+q_{pm}=-\frac{\partial }{\partial x}\left(N_{xx}\frac{\partial w}{\partial x}\right)-\frac{\partial }{\partial y}\left(N_{xy}\frac{\partial w}{\partial x}\right)\;-\frac{\partial }{\partial x}\left(N_{xy}\frac{\partial w}{\partial y}\right)-\frac{\partial }{\partial y}\left(N_{yy}\frac{\partial w}{\partial y}\right).$$

Here, *q_pm_* represents the external loading induced by the polymer matrix, which is obtained by

(20) $$q_{pm}=-k_{pm}^{\left(1\right)}w+k_{pm}^{\left(2\right)}\frac{\partial^{2}w}{\partial x^{2}}+k_{pm}^{\left(2\right)}\frac{\partial^{2}w}{\partial y^{2}},$$

where $k_{pm}^{\left(1\right)}$ and $k_{pm}^{\left(2\right)}$ stands for the Winkler and shear elastic coefficients of the polymer matrix. Substituting Equations (12), (13) and (20) into Equations (17)–(19), one obtains

(21) $$\begin{array}{l} {\tilde{K}}_{11}\frac{\partial^{2}u}{\partial x^{2}}+2{\tilde{K}}_{16}\frac{\partial^{2}u}{\partial x\partial y}+{\tilde{K}}_{66}\frac{\partial^{2}u}{\partial y^{2}}+{\tilde{K}}_{16}\frac{\partial^{2}v}{\partial x^{2}}+\left({\tilde{K}}_{12}+{\tilde{K}}_{66}\right)\frac{\partial^{2}v}{\partial x\partial y}+{\tilde{K}}_{26}\frac{\partial^{2}v}{\partial y^{2}}\\ -\left[{\tilde{Q}}_{11}\frac{\partial^{3}w}{\partial x^{3}}+3{\tilde{Q}}_{16}\frac{\partial^{3}w}{\partial x^{2}\partial y}+\left({\tilde{Q}}_{12}+2{\tilde{Q}}_{66}\right)\frac{\partial^{3}w}{\partial x\partial y^{2}}+{\tilde{Q}}_{26}\frac{\partial^{3}w}{\partial y^{3}}\right]=0, \end{array}$$

(22) $$\begin{array}{l} {\tilde{K}}_{16}\frac{\partial^{2}u}{\partial x^{2}}+\left({\tilde{K}}_{12}+{\tilde{K}}_{66}\right)\frac{\partial^{2}u}{\partial x\partial y}+{\tilde{K}}_{26}\frac{\partial^{2}u}{\partial y^{2}}+{\tilde{K}}_{66}\frac{\partial^{2}v}{\partial x^{2}}+2{\tilde{K}}_{26}\frac{\partial^{2}v}{\partial x\partial y}+{\tilde{K}}_{22}\frac{\partial^{2}v}{\partial y^{2}}\\ -\left[{\tilde{Q}}_{16}\frac{\partial^{3}w}{\partial x^{3}}+\left({\tilde{Q}}_{12}+2{\tilde{Q}}_{66}\right)\frac{\partial^{3}w}{\partial x^{2}\partial y}+3{\tilde{Q}}_{26}\frac{\partial^{3}w}{\partial x\partial y^{2}}+{\tilde{Q}}_{22}\frac{\partial^{3}w}{\partial y^{3}}\right]=0, \end{array}$$

(23) $$\begin{array}{l} {\tilde{Q}}_{11}\frac{\partial^{3}u}{\partial x^{3}}+3{\tilde{Q}}_{16}\frac{\partial^{3}u}{\partial x^{2}\partial y}+\left({\tilde{Q}}_{12}+2{\tilde{Q}}_{66}\right)\frac{\partial^{3}u}{\partial x\partial y^{2}}+{\tilde{Q}}_{26}\frac{\partial^{3}u}{\partial y^{3}}\\ +{\tilde{Q}}_{16}\frac{\partial^{3}v}{\partial x^{3}}+\left({\tilde{Q}}_{12}+2{\tilde{Q}}_{66}\right)\frac{\partial^{3}v}{\partial x^{2}\partial y}+3{\tilde{Q}}_{26}\frac{\partial^{3}v}{\partial x\partial y^{2}}+{\tilde{Q}}_{22}\frac{\partial^{3}v}{\partial y^{3}}\\ -\left[{\tilde{S}}_{11}\frac{\partial^{4}w}{\partial x^{4}}+4{\tilde{S}}_{16}\frac{\partial^{4}w}{\partial x^{3}\partial y}+2\left({\tilde{S}}_{12}+2{\tilde{S}}_{66}\right)\frac{\partial^{4}w}{\partial x^{2}\partial y^{2}}+4{\tilde{S}}_{26}\frac{\partial^{4}w}{\partial x\partial y^{3}}+{\tilde{S}}_{22}\frac{\partial^{4}w}{\partial y^{4}}\right]\\ -k_{pm}^{\left(1\right)}w+k_{pm}^{\left(2\right)}\frac{\partial^{2}w}{\partial x^{2}}+k_{pm}^{\left(2\right)}\frac{\partial^{2}w}{\partial y^{2}}-{\left(e_{0}l_{c}\right)}^{2}\left(-k_{pm}^{\left(1\right)}\frac{\partial^{2}w}{\partial x^{2}}-k_{pm}^{\left(1\right)}\frac{\partial^{2}w}{\partial y^{2}}\right.\\ \left.+k_{pm}^{\left(2\right)}\frac{\partial^{4}w}{\partial x^{4}}+2k_{pm}^{\left(2\right)}\frac{\partial^{4}w}{\partial x^{2}\partial y^{2}}+k_{pm}^{\left(2\right)}\frac{\partial^{4}w}{\partial y^{4}}\right)\\ +N_{xx}^{r}\left[\frac{\partial^{2}w}{\partial x^{2}}-{\left(e_{0}l_{c}\right)}^{2}\left(\frac{\partial^{4}w}{\partial x^{4}}+\frac{\partial^{4}w}{\partial x^{2}\partial y^{2}}\right)\right]\\ +N_{yy}^{r}\left[\frac{\partial^{2}w}{\partial y^{2}}-{\left(e_{0}l_{c}\right)}^{2}\left(\frac{\partial^{4}w}{\partial x^{2}\partial y^{2}}+\frac{\partial^{4}w}{\partial y^{4}}\right)\right]\\ +2N_{xy}^{me}\left[\frac{\partial^{2}w}{\partial x\partial y}-{\left(e_{0}l_{c}\right)}^{2}\left(\frac{\partial^{4}w}{\partial x^{3}\partial y}+\frac{\partial^{4}w}{\partial x\partial y^{3}}\right)\right]=0, \end{array}$$

where $N_{xy}^{me}$ is the shear in-plane load exerted on the hybrid nanoplate (see Figure 1).

## 3. Numerical Calculation Based on DQ Method

A numerical solution is constructed on the basis of the differential quadrature (DQ) method. According to this numerical method, a derivative of a given function in a differential equation is replaced by the following relations [63]

(24) $$\begin{array}{l} \frac{\partial^{p}\bar{u}}{\partial {\bar{x}}^{p}}=\sum_{k=1}^{n_{x}}A_{i,k}^{\left(p\right)}{\bar{u}}_{k,j},\;\;\;\;\;\frac{\partial^{q}\bar{u}}{\partial {\bar{y}}^{q}}=\sum_{k=1}^{n_{y}}B_{j,k}^{\left(q\right)}{\bar{u}}_{i,k},\\ \frac{\partial^{\left(p+q\right)}\bar{u}}{\partial {\bar{x}}^{p}\partial {\bar{y}}^{q}}=\sum_{k=1}^{n_{x}}\sum_{l=1}^{n_{y}}A_{i,k}^{\left(p\right)}B_{j,l}^{\left(q\right)}{\bar{u}}_{k,l}, \end{array}$$

where ${\bar{u}}_{i,k}$ is the function value at point $\left({\bar{x}}_{i},{\bar{y}}_{k}\right)$ in which $\langle \bar{u},\bar{x},\bar{y}\rangle =\langle u/h,x/a,y/b\rangle$; the number of mesh points across the length and width are respectively denoted by *n_x_* and *n_y_*; $A_{i,k}^{\left(r\right)}$ and $B_{j,k}^{\left(r\right)}$ are the *r*th-order weighting coefficients. For the first-order ones, we have

(25) $$\begin{array}{l} A_{ij}^{\left(1\right)}=\left\{\begin{array}{c} \frac{P^{\left(1\right)}({\bar{x}}_{i})}{\left({\bar{x}}_{i}-{\bar{x}}_{j})P^{\left(1\right)}({\bar{x}}_{j}\right)}\;\;\;\;\;for\;i\neq j\\ -\sum_{j=1(j\neq i)}^{n_{x}}A_{ij}^{\left(1\right)}\;\;\;\;\;\;\;\;\;for\;i=j \end{array}\right.\;,\\ B_{ij}^{\left(1\right)}=\left\{\begin{array}{c} \frac{P^{\left(1\right)}({\bar{y}}_{i})}{\left({\bar{y}}_{i}-{\bar{y}}_{j})P^{\left(1\right)}({\bar{y}}_{j}\right)}\;\;\;\;\;for\;i\neq j\\ -\sum_{j=1(j\neq i)}^{n_{y}}B_{ij}^{\left(1\right)}\;\;\;\;\;\;\;\;\;for\;i=j \end{array}\right.\;, \end{array}$$

in which P and $P^{\left(1\right)}$ are obtained as

(26) $$P(\bar{z})=\prod_{j=1}^{n_{z}}\left(\bar{z}-{\bar{z}}_{j}\right),\;P^{\left(1\right)}({\bar{z}}_{k})=\prod_{j=1(j\neq k)}^{n_{z}}\left({\bar{z}}_{k}-{\bar{z}}_{j}\right).$$

Here $\bar{z}=\bar{x}$ or $\bar{y}$. It should be noted that the higher-order weighting coefficients of the DQ method are expressed as

(27) $$\begin{array}{l} A_{i,j}^{\left(2\right)}=\sum_{k=1}^{n_{x}}A_{i,k}^{\left(1\right)}A_{k,j}^{\left(1\right)},\;\;A_{i,j}^{\left(3\right)}=\sum_{k=1}^{n_{x}}A_{i,k}^{\left(2\right)}A_{k,j}^{\left(1\right)},\;\;A_{i,j}^{\left(4\right)}=\sum_{k=1}^{n_{x}}A_{i,k}^{\left(2\right)}A_{k,j}^{\left(2\right)},\\ B_{i,j}^{\left(2\right)}=\sum_{k=1}^{n_{y}}B_{i,k}^{\left(1\right)}B_{k,j}^{\left(1\right)},\;\;B_{i,j}^{\left(3\right)}=\sum_{k=1}^{n_{y}}B_{i,k}^{\left(2\right)}B_{k,j}^{\left(1\right)},\;\;B_{i,j}^{\left(4\right)}=\sum_{k=1}^{n_{y}}B_{i,k}^{\left(2\right)}B_{k,j}^{\left(2\right)}. \end{array}$$

For the sake of accuracy and according to the Gauss-Chebyshev–Lobatto law [63], the following distribution is utilized for the grid points

(28) $$\begin{array}{l} {\bar{x}}_{k}=0.5-0.5\cos\left(\Phi_{k}^{\left(x\right)}\right),\;\;\Phi_{k}^{\left(x\right)}=\frac{\pi \left(k-1\right)}{n_{x}-1},\;\;\mathrm{for}\;k=1,2,\ldots ,n_{x},\\ {\bar{y}}_{k}=0.5-0.5\cos\left(\Phi_{k}^{\left(y\right)}\right),\;\;\Phi_{k}^{\left(y\right)}=\frac{\pi \left(k-1\right)}{n_{y}-1},\;\;\mathrm{for}\;k=1,2,\ldots ,n_{y}. \end{array}$$

In view of Equation (24) and using the direct approach for the implementation of boundary conditions, one obtains

(29) $$\left(\left[\Xi \right]-{\bar{N}}_{xy}^{me}\left[\Theta \right]\right)\left\{\bar{d}\right\}=0,$$

where ${\bar{N}}_{xy}^{me}$ denotes the non-dimensional buckling load, which is defined by ${\bar{N}}_{xy}^{me}=N_{xy}^{me}a^{2}/{\tilde{S}}_{11}$; $\left\{\bar{d}\right\}$ is the displacement matrix. The critical shear buckling force is obtained from Equation (29) through an eigenvalue solution procedure implemented in Python (version 3.12.12).

## 4. Results

**Figure 2:**
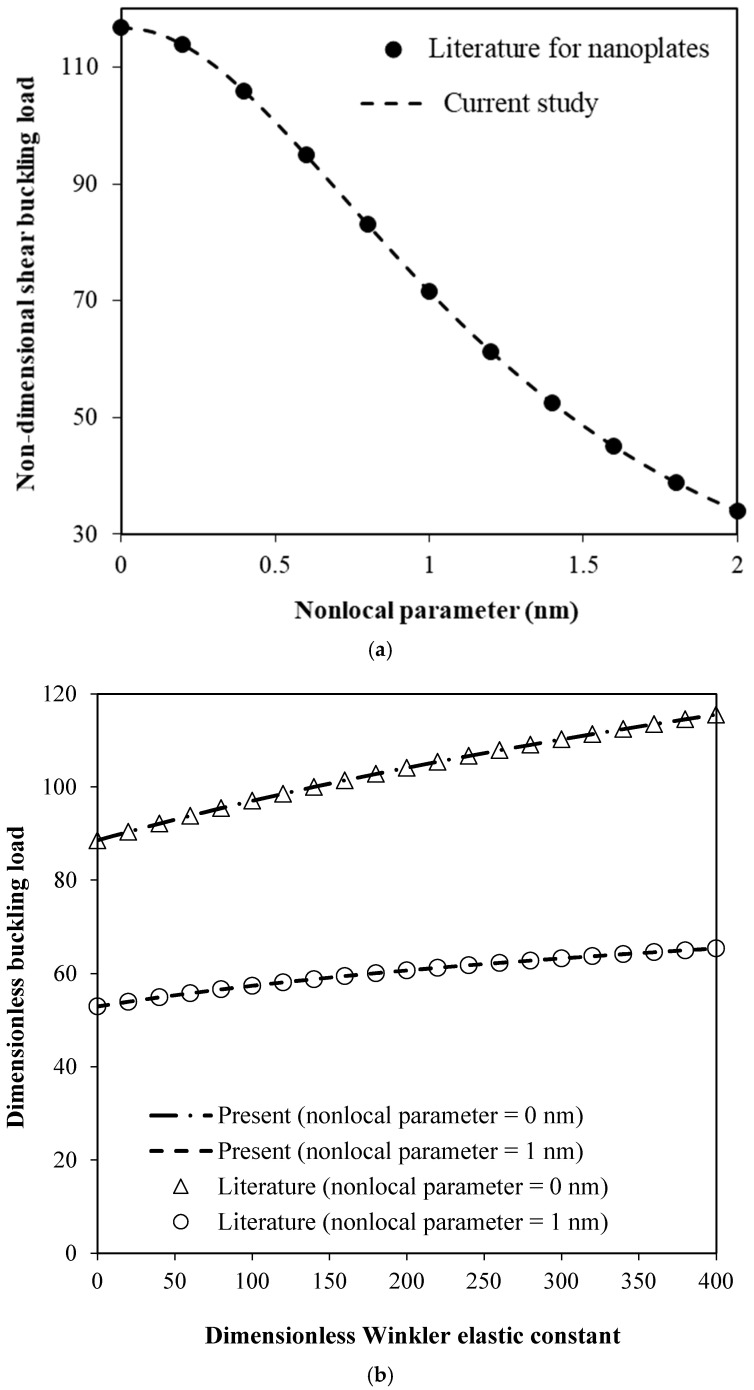
(**a**) Validation of the proposed nonlocal continuum model through comparison with nanoplate results reported in Ref. [25] for the shear buckling response for various nonlocal parameters from 0 to 2 nm. (**b**) Validation study through comparison with the results reported in Ref. [25] for the shear buckling of single-layered nanoplates embedded in a surrounding elastic medium for various dimensionless Winkler elastic constants from 0 to 400.

The shear buckling analysis of SMA nanofiber-reinforced hybrid plates is investigated in the following section. First of all, the accuracy of the proposed continuum model is shown in Figure 2a by comparing the results of this methodology with those reported for typical nanoplates [25]. The elastic constants of the polymer matrix are taken as ${\bar{k}}_{pm}^{\left(1\right)}=100$ and ${\bar{k}}_{pm}^{\left(2\right)}=10$ where ${\bar{k}}_{pm}^{\left(1\right)}=k_{pm}^{\left(1\right)}a^{4}/{\tilde{S}}_{11}$ and ${\bar{k}}_{pm}^{\left(2\right)}=k_{pm}^{\left(2\right)}a^{2}/{\tilde{S}}_{11}$ are, respectively, the non-dimensional Winkler and shear elastic constants. A simply supported square nanoplate of thickness 0.34 nm and length 10 nm is considered. The elastic properties of the nanoplate are considered as *E*_11_ = 1765 GPa, *E*_22_ = 1588 GPa, *v*_12_ = 0.3 and *v*_21_ = 0.27. From Figure 2a, it is clearly seen that the results of the current study for the shear buckling of nanoplates are in excellent agreement with those reported in the literature. Further verification is conducted by comparing the results reported in Ref. [25] for the shear buckling of single-layered nanoplates embedded in a surrounding elastic medium with those of the present nonlocal model in Figure 2b. SMA effects are ignored while the Winkler effect of the surrounding elastic medium is taken into consideration. However, the shear constant of the surrounding elastic medium is assumed to be zero. The verification study is performed for various values of the dimensionless Winkler elastic constant in the range of 0 to 400. The results are plotted for both classical plates (nonlocal parameter = 0) and nanoplates (nonlocal parameter = 1 nm). An excellent agreement is found in Figure 2b between the results of the present model and those of the previous study for all different cases, demonstrating the accuracy of the present approach when the effect of the surrounding elastic medium is considered.

A composite ultrasmall plate composed of three square nanoplates with a length of 100 nm and a thickness of 3 nm is taken into account. Poisson’s ratio and Young’s modulus of SMA nanofibers are 0.3 and 30 GPa, respectively. The volume fraction of the SMA nanofibers in the middle layer is 0.3, while for the typical nanofibers in the top and bottom plates, it is set to 0.1. The elasticity moduli of typical nanofibers and the nanoplate are assumed as 39 and 3.44 GPa, respectively [64]; moreover, their Poisson’s ratios are, respectively, considered as 0.28 and 0.35. The orientation of SMA nanofibers is described by $\psi_{SMA}=0$ while the typical nanofibers in the top and bottom plates are placed as $\psi_{tnf}=90$ in which ‘*tnf*’ denotes typical nanofibers. The boundary conditions of the reinforced hybrid nanoscale system are simply supported. In this section, unless otherwise mentioned, the mechanical and geometrical properties listed above are assumed for the hybrid nanoscale system.

In a recent experimental study [65], the practical range of recovery stress values for a type of SMA nanofibers has been reported to vary approximately between 0 and 900 MPa, depending on alloy composition, phase transformation features and temperature conditions. In the present study, the recovery stresses of the SMA nanofibers are taken in the range of 0 to 300 MPa, which are all within the experimentally reported practical range [65]. Therefore, the chosen interval represents realistic operating scenarios for SMA-reinforced hybrid small-scale structures. The selected recovery stress values ensure that the findings of this modelling approach about the shear instability control are physically meaningful and expected in practical cases.

**Figure 3:**
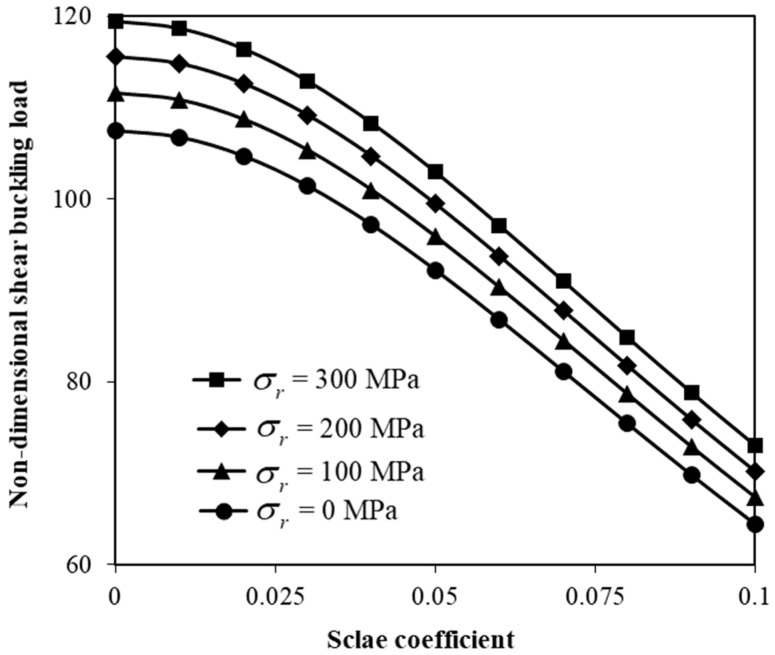
Adjustment of shear instability force by the recovery stress.

**Figure 4:**
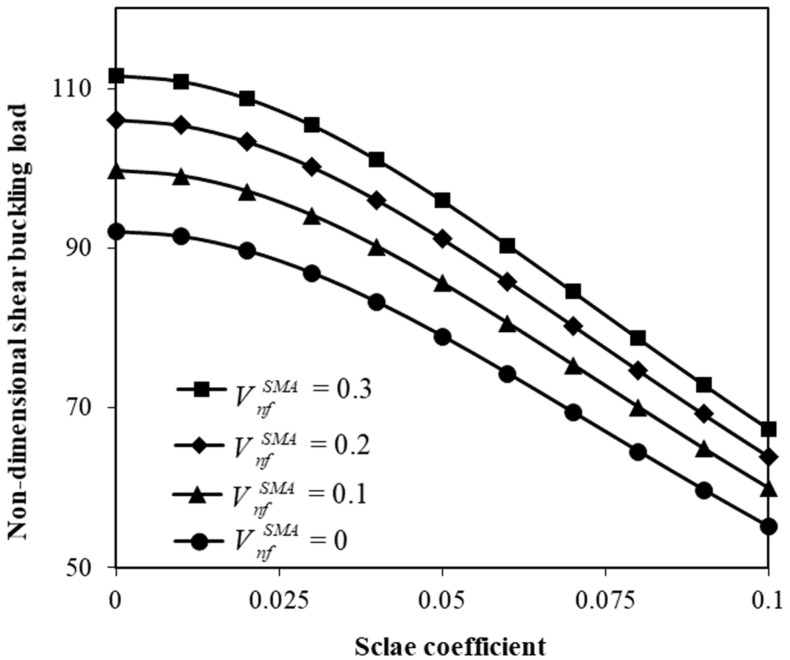
Adjustment of shear instability force by the SMA volume fraction.

Figure 3 depicts the variation in the shear buckling force versus the size parameter $(e_{0}l_{c}/a)$ for various recovery stresses. The shear buckling force decreases with increasing scale coefficient due to the effect of the scale nonlocality. In addition, the shear buckling force is higher for higher recovery stresses. The influence of the SMA volume fraction on the shear force is also indicated in Figure 4. As the SMA volume fraction increases, the shear buckling load notably increases. The ratio of the volume fraction of the typical nanofibers in the top and bottom layers to that of the mid-layer is one-third. Moreover, the SMA recovery stress is set to 100 MPa.

The nonlocal formulation helps to take into account the effects of long-range molecular and sub-molecular interactions on the mechanical response. An increase in nonlocal effects results in a reduction in the stiffness of the structure due to enhanced small-scale long-range interactions. This stiffness reduction consequently decreases the critical shear instability load, leading to a softening response. This behaviour becomes more evident at smaller scales, where stress nonlocality dominates the mechanical response. In contrast, SMA-nanofiber reinforcement introduces a stiffness hardening behaviour. This hardening behaviour is mainly due to the recovery stresses generated during phase transformation, which improve the effective rigidity of the hybrid structure and enhance resistance to shear loads. The overall instability response, therefore, is governed by a combination of these two behaviours: (1) nonlocal effects that decrease the critical shear instability load by reducing effective stiffness, and (2) SMA recovery stresses counteract this effect by increasing structural resistance.

**Figure 5:**
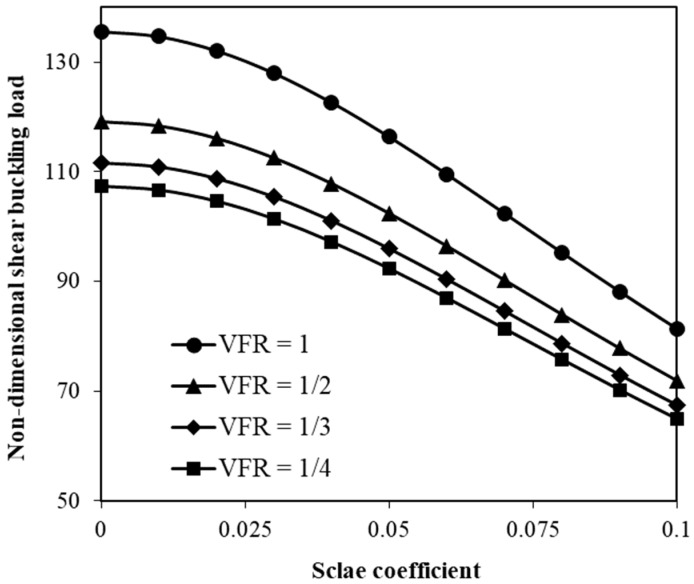
Adjustment of shear instability force by volume fraction ratio.

**Figure 6:**
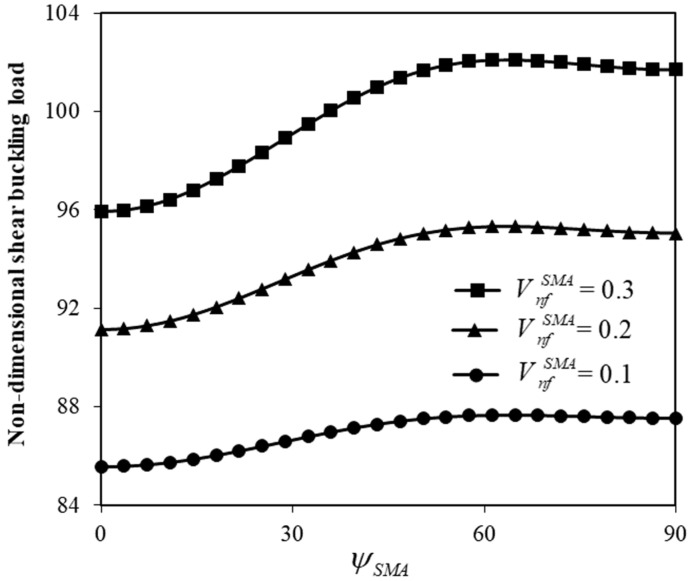
Adjustment of shear instability force by the angle at which SMA nanofibers are placed, for different SMA volume fractions.

Figure 5 shows the influence of the ratio of SMA volume fractions to those of typical ones on the shear buckling. Reducing the volume fraction ratio (VFR) results in a notable decrease in the shear buckling force. Furthermore, Figure 6 illustrates the change in the shear buckling force versus the angle at which SMA nanofibers are placed. Different SMA volume fractions are taken into consideration. The nonlocal scale coefficient of the hybrid system is set to 0.05. Increasing the angle of the SMA orientation leads to an increase in the shear buckling load, which is followed by a slight decrease that occurs at a certain angle between 60° and 65°. The non-monotonic trend in the effect of nanofiber orientation angle on the shear instability of the hybrid nanoplate is rooted in the fact that there is a significant variation in the stress transmission mechanism between the nanofibers and their surrounding plate. When the orientation angle is small, SMA nanofibers mainly contribute to axial stiffness, leading to less resistance to shear deformation. As the orientation angle increases, a larger component of the recovery stress and fiber stiffness is projected along the shear direction, improving the effective shear rigidity of the hybrid plate. This results in an increase in the critical shear instability load up to an optimal point that occurs approximately somewhere between 60° and 65°. Beyond this point, further increase in the orientation angle decreases the effective contribution of fibers to the shear-force carrying capacity of the system. This leads to a slight reduction in the critical shear instability load.

**Figure 7:**
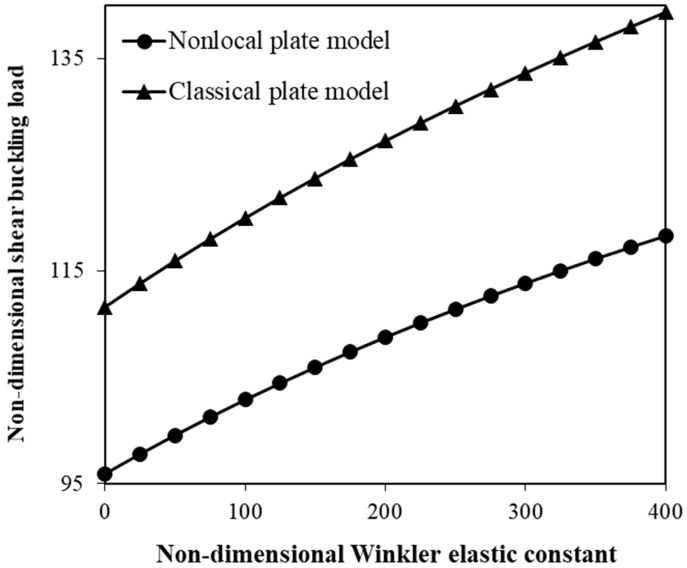
Adjustment of shear instability force by the Winkler elastic constant for classical and nonlocal plate models.

Figure 7 is plotted to show the change in the shear buckling force of the nanoplate with simply supported edges versus the Winkler constant of the elastic medium for both the local elasticity theory and the nonlocal plate model. The volume fraction of SMA nanofibers is 0.3, while the volume fraction of typical nanofibers is 0.1. The orientation of SMA nanofibers is described by $\psi_{SMA}=0,$ whereas the typical nanofibers are placed as $\psi_{tnf}=90$. The effect of the shear elastic constant is not taken into account. A value of 0.05 is considered for the scale coefficient in the nonlocal plate model, while the scale coefficient is set to zero in the classical model. From Figure 7, it is seen that increasing the Winkler elastic constant increases the shear buckling force. In addition, it is found that the classical model overestimates the critical shear force.

**Figure 8:**
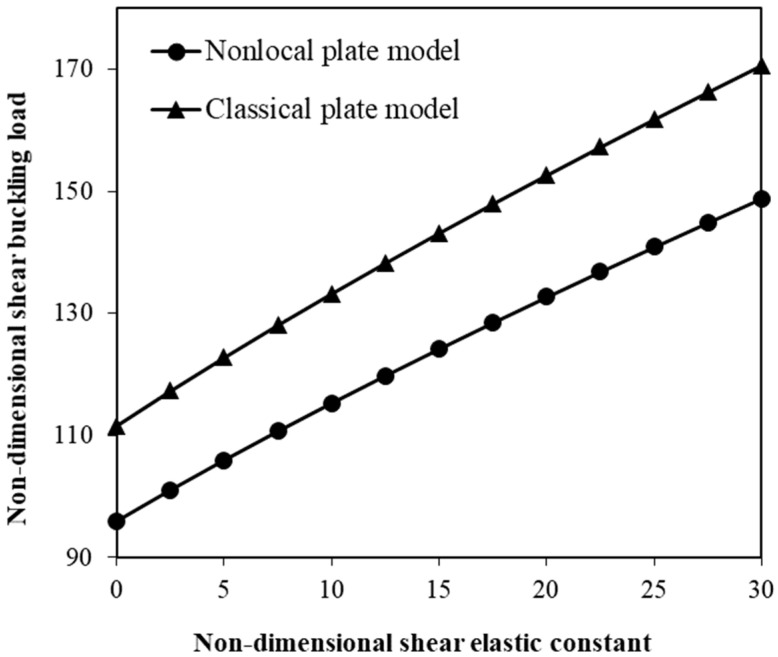
Adjustment of shear instability force by the shear elastic constant for classical and nonlocal plate models.

**Figure 9:**
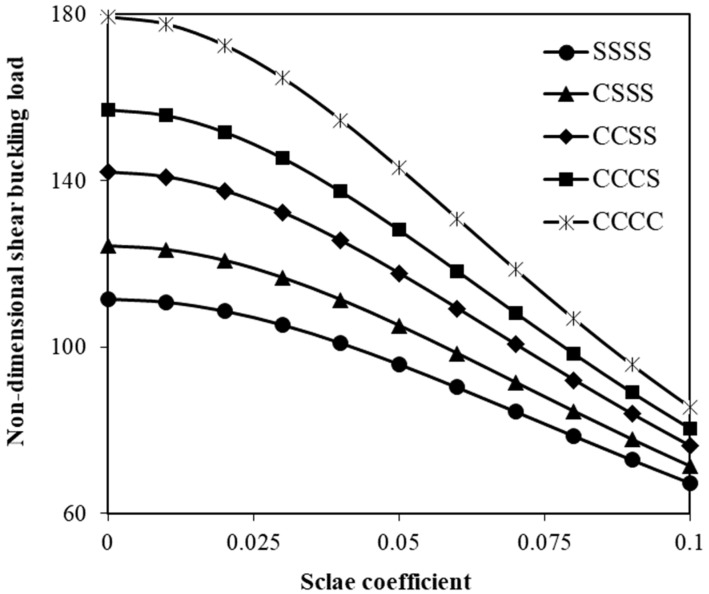
Adjustment of shear instability force by changing the boundary condition of the small-scale hybrid plate; scale effects are also illustrated by changing the size parameter on the horizontal axis.

The effect of the shear elastic constant of the medium is also indicated in Figure 8. The Winkler elastic constant is assumed to be zero in this figure to emphasize the role of the surrounding medium’s shear elastic constant in the shear instability response of the system. As the shear elastic constant is increased, the critical shear force gradually increases. Figure 9 shows the change in the critical shear force of the hybrid nanoplate versus the size parameter for different edge conditions. As the boundary condition of the nanosystem becomes stiffer, the shear buckling load significantly increases. This enhancement pattern is more pronounced at smaller values of the scale coefficient.

The influence of boundary conditions on the critical shear instability load can be connected to the practical configurations, which are often found in MEMS/NEMS devices and polymer-embedded multilayer structures. Particularly, the enhancement of shear loading capacity observed for clamped edges can be implemented by a combination of surrounding substrates, bonding layers and encapsulating polymers. In many microscale and nanoscale electromechanical devices, the fundamental structural elements of the system are partially or fully constrained by surrounding substrates, bonding layers, or encapsulating polymers. These tools can effectively impose clamped or near-clamped boundary conditions. Such constraints restrict rotational and transverse displacements at the system edges, resulting in improved structural stiffness and higher critical instability loads. Therefore, the observed increase in the critical shear load of the hybrid system subject to clamped boundary conditions represents practical scenarios in MEMS/NEMS devices such as small-scale sensing systems, actuators, and generators.

## 5. Discussion

The variation in the shear buckling force with the size parameter linked to stress nonlocalities has been studied by taking into account different recovery stress levels (Figure 3). The results indicate that the shear buckling force is decreased when a higher size coefficient is adopted. This phenomenon is associated with the enhancement of nonlocal influences that soften the nanostructure and reduce the flexural rigidity of the hybrid nanosystem [41]. As a result, the critical shear buckling force is decreased. Moreover, a higher recovery stress leads to enhanced shear buckling loads since the hybrid nanosystem has an internal pre-stress field that helps it to recover more rapidly. This also results in additional membrane stiffening and thus greater resistance to an applied shear force.

The influence of the SMA volume fraction on the shear buckling force has also been investigated (refer to Figure 4). The numerical data would suggest that the critical shear buckling force increases significantly when a higher volume fraction of SMA nanofibers is used in the manufacturing process of the hybrid system. The reason is rooted in the enhanced flexural stiffness and recovery stresses introduced by the SMA reinforcement that generate a kind of internal stabilizing stress within the hybrid nanoplate. As a consequence, the increased SMA portion improves the resistance of the structure to shear deformation and delays the onset of shear instability.

In addition to the volume fraction of SMA nanofibers (Figure 4 and Figure 5), the geometrical angle at which these nanofibers are placed also plays a key role in the control of the shear instability of small-scale hybrid nanoscale plates (Figure 6). As the angle of the SMA nanofiber orientation increases from 0 to 90 degrees, the shear buckling load first increases. This increase is followed by a slight reduction in the shear buckling force, which is observed at a certain angle between 600 and 650. Beyond this point, the influence of the SMA nanofiber angle becomes less pronounced, and the change in the shear buckling force with the SMA orientation angle flattens.

The surrounding polymer matrix acts as another important controlling factor in the shear instability behaviour of the hybrid nanoscale system composed of SMA nanofibers and nanoplates (Figure 7 and Figure 8). Particularly, two polymer-matrix parameters are found to be impactful: (1) the Winkler elastic constant, and (2) the shear elastic constant of the surrounding medium. Changes in the shear buckling force with variations in these parameters are investigated in this study. The numerical calculations show that increasing the Winkler elastic constant leads to a higher critical shear buckling force. This is because an enhanced normal stiffness of the elastic medium provides stronger elastic restraint against the transverse deflection of the hybrid nanoplate under external shear loading. Furthermore, an increase in the shear elastic constant of the medium results in a gradual increase in the critical shear force as the surrounding medium can exhibit higher resistance to in-plane shear deformation, thus improving the overall stability of the hybrid nanosystem and delaying the onset of shear buckling. These findings are consistent with previous studies on the scale-dependent mechanical behaviour of small-scale structures embedded in an elastic medium [50,66].

The variation in the critical shear buckling force of nanoplates reinforced by SMA nanofibers with the size parameter has been analyzed for different edge conditions (Figure 9). The results show that stiffer boundary conditions lead to a significant increase in the shear buckling load. This observation is due to the fact that enhancing edge constraints restricts transverse deformation and improves the effective structural stiffness of the hybrid nanosystem. This strengthening effect is more pronounced at smaller values of the scale coefficient, where nonlocal softening effects are weaker and boundary restraints play a more dominant role in the shear instability behaviour. The magnitude of this increase in the shear buckling force resulting from stiffer boundary conditions can be adjusted by changing the number of edges with clamped boundary constraints.

It should be noted that the effectiveness of SMA nanofibers at small scales is related to the size-dependent nature of martensitic phase transformations. Previous experimental studies on Ti_2_NiCu alloy nanostructures have shown that the martensitic transformation temperature decreases significantly with decreasing structural thickness, and that the transformation may be completely suppressed below a critical thickness of approximately 20 nm [67]. This behaviour highlights an important physical limitation in the application of SMA-nanofiber reinforcements, as the shape memory effect and its associated recovery stresses may significantly decrease or even disappear when structural dimensions fall below a specific size. Although the present size-dependent continuum formulation is applicable across a wide range of dimensions and it reduces to the classical continuum model when the nonlocal parameter vanishes, the practical implementation of SMA nanofibers should consider dimensional regimes in which martensitic transformations remain active. The inclusion of such size-limit considerations provides useful guidance for the design and optimization of SMA nanofiber-reinforced nanomechanical systems, particularly where efficient instability control is required. It is also important to note that this specific thickness (i.e., 20 nm) is only applicable to Ti_2_NiCu alloy nanostructures [67], and for other types of SMA nanostructures, this size might be different. Experimental measurement of this critical thickness size is crucial before the practical use of any SMA nanostructures for control purposes at nanoscales.

## 6. Conclusions

A coupled modified continuum model was presented to analyze the ability of SMA nanofibers to control the shear instability of hybrid small-scale plates embedded in a polymer matrix. To consider the scale effect, the Eringen theory of elasticity was successfully utilized. Application of the Brinson theory and the principle of virtual work, as well as the nonlocal elasticity, led to the coupled scale-dependent equations of the hybrid plate. The DQ approach was then applied to the coupled differential equations so as to obtain a numerical solution. The numerical results were obtained for different edge conditions, including simply supported, clamped, and their combination along various edges of the small-scale plate. The shear instability response of small-scale plates can be well controlled by using SMA nanofibers implemented into the plate structure. The shear instability force of hybrid plates decreases with increasing the scale coefficient due to the decreasing influence of stress nonlocality on the structural rigidity at small scales. The critical shear force is higher for higher recovery stresses. The shear force notably increases when the SMA volume fraction increases. The numerical results indicate that reducing the VFR leads to a noticeable decrease in the instability shear force of the hybrid plate. The orientation of SMA nanofibers can also be adjusted to control the shear instability of hybrid small-scale plates. Increasing the Winkler constant of the surrounding polymer matrix increases the shear instability force. Additionally, according to the results of this study, it can be concluded that making the edges of the hybrid small-scale plate stiffer yields a significant increase in the shear instability force.

## Data Availability

The original contributions presented in this study are included in the article. Further inquiries can be directed to the corresponding author.

## Abbreviations

The following abbreviations are used in this manuscript:

SMA	Shape Memory Alloy
NEMS	Nano-Electro-Mechanical Systems
MEMS	Micro-Electro-Mechanical Systems
CNT	Carbon Nanotube
DQ	Differential Quadrature
NPs	Nanoplates
NFs	Nanofibers
TNFs	Typical Nanofibers
VFR	Volume Fraction Ratio
